# Coccydynia relieved by a tarsal tunnel block: a case series

**DOI:** 10.1186/s13256-019-2275-5

**Published:** 2019-11-21

**Authors:** Amjid Hammodi

**Affiliations:** Baghdad, Iraq

**Keywords:** Coccydynia, Tarsal tunnel block, Gate C injection, Anatomical gates of sodium channel blockers, Retrograde analgesia

## Abstract

**Background:**

This case series describes, for the first time, to the author’s knowledge, a novel treatment for coccydynia. Tarsal tunnel block with lignocaine only brought relief of chronic coccydynia lasting more than 6 months in three patients. The author adopts the theory that the myelin sheath of the posterior tibial nerve will convey the lipid-soluble lignocaine upward toward the dorsal root ganglia and the nerve roots of the lumbar spine through the uninterrupted myelin sheath, which is itself mainly formed of lipids. The author thinks that most coccyx pain is actually a radiating pain from the lumbar spine, which is not always apparent on magnetic resonance imaging of the lumbar spine. Certainly, the author acknowledges that large-scale studies need to be done to prove the efficacy of this new technique and to prove that the myelin sheath can convey the lignocaine chemical upward.

**Case presentation:**

Three Arab patients presented with chronic coccydynia of more than 6 months’ duration in whom conservative management had failed to control their symptoms. They had no past medical history of significance and no history of trauma. The results of physical examination of all of the patients were normal apart from tenderness on palpation of the coccyx. They all received local coccyx injection with steroids on two occasions, which failed to relieve their pain. One patient underwent manipulation under anesthesia, and one underwent coccygectomy with no pain relief. Magnetic resonance imaging results were reported to be normal in two of them, whereas the other one had a prolapsed disc at the L4/L5 level. The three patients described pain relief 30 minutes after tarsal tunnel block with lignocaine only lasting more than 6 months. All patients had heel anesthesia 15 minutes after the tarsal tunnel injection, which lasted only 1 hour.

**Conclusions:**

Tarsal tunnel block with lignocaine can relieve coccyx pain for a long time. Tarsal tunnel block can be done to achieve heel anesthesia before injection of lignocaine into the plantar fascia in patients with plantar fasciitis.

## Introduction

In 30% of patients with coccydynia, the cause of pain cannot be found (idiopathic coccygodynia) [1]. I describe, for the first time, to my knowledge, the successful treatment of coccydynia with tarsal tunnel block using lignocaine only, without steroids, and the pain relief lasting over 6 months that was achieved in three patients case series.

I believe that after taking a careful history and performing proper examination and pertinent investigations, the main cause of coccyx pain is actually pain radiating from the location of lumbar spine disc disease, but still other causes should be ruled out carefully.

**Fig. 1:**
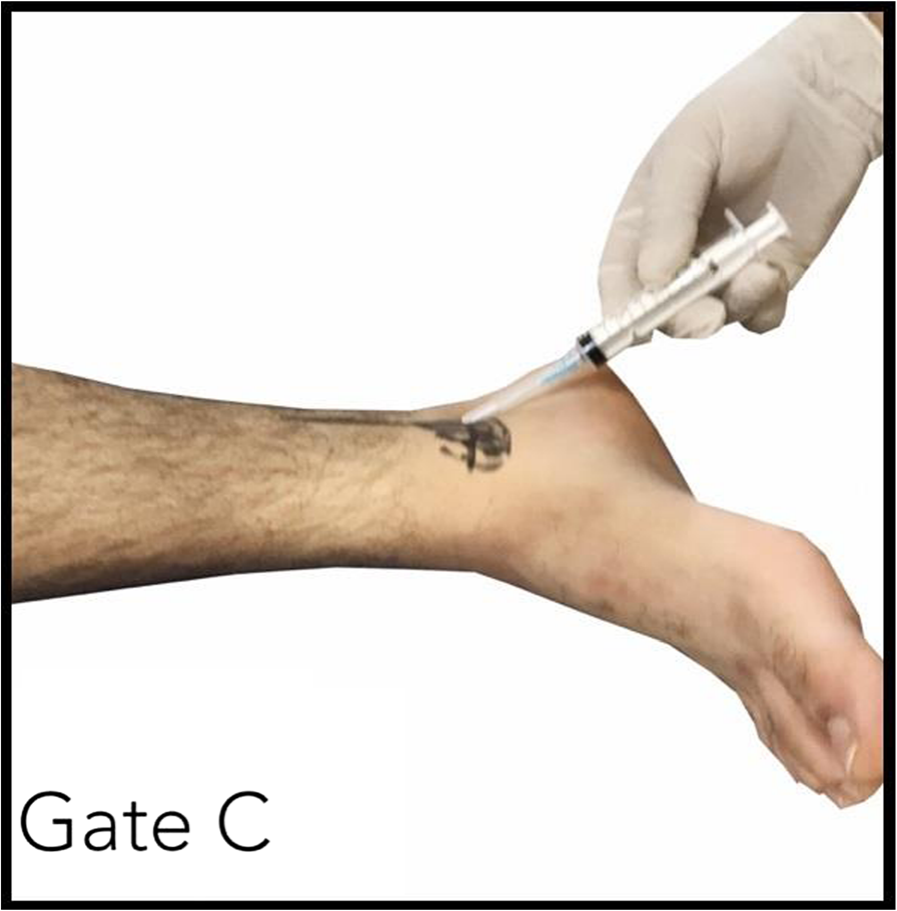
Tarsal tunnel injection (Gate C) with lignocaine only

I describe in this case series, for the first time, to my knowledge, successful treatment of coccydynia with tarsal tunnel block using lignocaine only (Fig. 1). This injection has been named *gate C injection* by the author, and gate C is one of the anatomical gates of sodium channel blockers discovered by the author [2].

After injecting the tarsal tunnel with lignocaine, the heel and almost all of the foot will go numb within 15 minutes, and this particular numbness in the heel is the indication that the injection was successful in the tarsal tunnel. This numbness should last around 1 hour. Around 30 minutes after the injection, the patient will confirm the relief of pain.

## Case presentation

### Patient 1

Patient 1 was a 45-year-old Arab man with no significant past medical, family, or psychosocial history who had been experiencing coccydynia for the past 10 years. The result of his physical examination was normal apart from tenderness on palpation of the coccyx.

He had received steroid injections to his coccyx on two occasions, which had failed to relieve his pain.

Magnetic resonance imaging (MRI) of his lumbar spine showed L4/L5 disc prolapse, and he had undergone microdiscectomy of his L4/L5 disc 5 years ago, which made the coccyx pain worse. The patient had undergone coccygectomy 3 years ago, which again made his coccyx pain worse.

The tarsal tunnel block, gate C, was performed without ultrasound guidance and was deemed successful when heel anesthesia was reported by the patient 15 minutes after the injection.

The injection volume was 10 ml (3 ml of 2% lignocaine HCl plus 7 ml of distilled water).

The patient received the injection at gate C on one side, and 30 minutes after the injection, he reported 80% pain relief. The patient was reviewed 6 months later, and he continued to live with 80% pain relief.

### Patient 2

Patient 2 was a 33-year-old Arab orthopedic doctor with no significant past medical, family, or psychosocial history who had a complaint of coccydynia of 1 year’s duration. The result of his physical examination was normal apart from tenderness on palpation of the coccyx.

The pain was very severe, preventing the patient from sitting up and affecting his daily activities.

The radiologist reported that the result of MRI of his lumbar spine was normal.

The patient had received steroid injections into his coccyx on two occasions, with one manipulation under anesthesia, but all had failed to relieve his pain.

The patient was due to undergo coccygectomy when he heard about the new sodium channel blocker injection gates.

Tarsal tunnel block, gate C, was performed without ultrasound guidance and was deemed successful when heel anesthesia was reported by the patient 15 minutes after the injection.

The injection volume was 10 ml (3 ml of 2% lignocaine HCl plus 7 ml of distilled water).

The patient reported complete pain relief 30 minutes after the injection, and his pain relief continued for the next 9 months.

### Patient 3

Patient 3 was a 36-year-old Arab man with no significant past medical, family, or psychosocial history who presented with a 9-month history of severe coccyx pain affecting his daily activities. He report no history of trauma.

The result of his physical examination was normal apart from tenderness on palpation of the coccyx.

He rated his pain severity as 10 on a visual analogue scale.

MRI was performed, and the result was reported as normal.

The patient received two coccyx steroid injections, which failed to relieve his pain. He then received bilateral tarsal tunnel block with lignocaine only seven months ago (at the time of writing this manuscript).

The patient reported complete pain relief 30 minutes after the injection, and his pain relief continued for the next 7 months.

## Discussion

The author adopts the theory that most coccyx pain is actually radiating pain coming from the location of lumbar spine disc disease.

How can a peripheral injection relieve pain when the cause of pain lies more proximally, as in this case series?

How can the effect of a simple lignocaine injection last for months when the half-life of lignocaine is only about 1 hour?

To understand that, the following must be understood: After nerve injury, hyperexcitability and spontaneous firing develop at the site of injury and also in the dorsal root ganglion cell bodies. This hyperexcitability results at least partly from accumulation of sodium channels at the site of injury. Pain is related to excited nerves and disturbance in voltage-gated sodium channels. The relationship between voltage-gated sodium channels and pain has been reported in many published studies [3–8].

Simply by administering lignocaine, which is a sodium channel blocker, the electrogenicity of nerves is being reset, like resetting a jammed laptop.

The local anesthetic molecule consists of three components: (1) lipophilic aromatic ring, (2) intermediate ester or amide chain, and (3) a terminal amine. The aromatic ring improves lipid solubility. The nerve membrane consists of a double lipid layer and a protein layer; therefore, the property of enhancing lipid solubility contributes to increased potency of the anesthetic agent because more of the available drug can diffuse through the membrane [9]. The myelin sheath is characterized by a high proportion of lipid (70–85%) [10].

Lignocaine will diffuse through the myelin sheath covering the nerve, and if a peripheral nerve is blocked, the diffusion will continue throughout the whole nerve blocking the sodium channels, which are accumulated at the site of injury.

The hypothesis is that diffusion will occur through the uninterrupted myelin sheath covering the main nerve and its branches, and by injecting one division of the main nerve, the lipid-based chemical will diffuse to the main nerve and its other branches.

Of course, the above-mentioned points need extensive research studies to prove or refute them, and the author suggests the use of contrast material, as is used in myelography, to prove that the chemicals can diffuse and travel proximally in the peripheral nerves. Contrast material, for example, can be injected into the tarsal tunnel and followed by radiology performed at various times to study this phenomenon. With this approach, a lot of knowledge could be gained in regard to nerve function, and the scope of that knowledge would be limitless.

## Conclusion

Tarsal tunnel injection with lignocaine can be done as a substitute to general anaesthesia in patients with plantar fasciitis requiring injection of the plantar fascia, usually with steroids, as the latter injection is very painful if it is done without anaesthesia.

## Data Availability

The datasets during and/or analyzed during the current study are available from the corresponding author on reasonable request.
